# Perinatal and gestational factors associated with Pavlik harness outcomes in developmental dysplasia of the hip: a retrospective cohort study

**DOI:** 10.3389/fped.2026.1938073

**Published:** 2026-09-28

**Authors:** Xinyue Liang, Boyuan Ma, Rong Li, Xinyi Jia, Zhiqiang You, Shuting Wang, Jianping Yang

**Affiliations:** 1 Department of Functional Examination, Tianjin Center for Women and Children’s Health, Tianjin, China; 2 Department of Pediatric Orthopedics, Tianjin Hospital, Tianjin, China; 3 Department of Children Health Administration, Tianjin Center for Women and Children’s Health, Tianjin, China

**Keywords:** developmental dysplasia of the hip, nomogram, Pavlik harness, perinatal and gestational factors, predictive model

## Abstract

**Introduction:**

Perinatal and gestational factors contribute to both the pathogenesis and prognosis of developmental dysplasia of the hip (DDH). However, data regarding the association between these factors and Pavlik harness treatment outcomes remain scarce. This study aimed to assess the associations between perinatal/gestational factors and Pavlik harness treatment outcomes, and to preliminarily establish a predictive model incorporating these factors to inform early treatment optimization for DDH.

**Methods:**

Perinatal and gestational data, along with treatment outcomes, were collected from children diagnosed with DDH through screening in Tianjin, China, between 2013 and 2023, who were treated with the Pavlik harness. All analyses were performed at the patient level. A multivariable logistic regression model was developed to identify perinatal and gestational factors independently associated with treatment outcomes. Receiver operating characteristic (ROC) curves were constructed to evaluate the model's discriminative ability, and a nomogram was developed for outcome prediction. Internal validation was performed using bootstrap resampling with 200 iterations.

**Results:**

Among 792 children (910 hips) included, low Graf classification, higher maternal education level, cephalic intrauterine position, parity > 1, maternal combined vitamin A and D supplementation during pregnancy and regular maternal milk consumption during pregnancy were significantly associated with higher treatment success rates. The overall prediction model, integrating Graf classification with the five perinatal and gestational factors, achieved a bootstrap-corrected C-index of 0.781, with significantly higher diagnostic accuracy than any single factor or alternative combination (AUC = 0.796, *P* < 0.05). In the Graf-stratified prediction models, the ROC curves for the five perinatal and gestational factors demonstrated acceptable discriminative ability (Stratum 1: C-index = 0.724; Stratum 2: C-index = 0.707). However, the model showed no predictive value in Graf type IV hips, where disease severity appeared to be the dominant determinant of treatment failure. Based on these findings, both overall and Graf-stratified nomograms were developed to predict Pavlik harness treatment outcomes in DDH.

**Conclusion:**

Several perinatal and gestational factors were independently associated with Pavlik harness treatment outcomes in DDH. The nomograms developed in this study may serve as preliminary decision-support tools for predicting treatment outcomes and facilitating the selection of more appropriate clinical management strategies, particularly for Graf types IIc and D/III. Given the observational study design, these findings should be interpreted as exploratory associations rather than causal determinants, and external validation is warranted before clinical implementation.

## 1 Introduction

Developmental dysplasia of the hip (DDH) encompasses a spectrum of anatomical abnormalities of the hip joint, characterized by deformities of the femoral head and acetabulum, a pathological relationship between these structures, and frequently associated capsular laxity (1). DDH represents a relatively common orthopedic condition in the pediatric population (2). Successful treatment of DDH depends critically on early, atraumatic, and stable reduction of the hip joint, which facilitates the proper development of the acetabulum and femoral head and mitigates the risk of avascular necrosis (AVN) (3).

For infants aged <6 months diagnosed with acetabular dysplasia or hip instability (Graf classification > type IIc) on ultrasound screening, the Pavlik harness is one of the commonly used treatment modalities (4). The Pavlik harness is a physiological, functional treatment modality. When applied, it positions the hip in 110° of flexion and 45–55° of abduction. In this flexed and abducted position—particularly during sleep when the hips abduct under gravity—the femoral head is naturally reduced into the acetabulum and maintained in a reduced position. Simultaneously, the harness allows the lower extremities to move freely within a safe range of flexion, extension, adduction, and abduction (5). Muscular activity around the hip joint generates rotational stress stimulation between the femoral head and acetabulum, which contributes to acetabular remodeling and development (6).

However, the success rate of harness treatment decreases with increasing patient age at presentation and greater severity of dislocation (7, 8). Furthermore, prolonged duration of harness therapy is associated with an increased risk of complications such as avascular necrosis of the femoral head (9, 10). Therefore, early prediction of Pavlik harness treatment outcomes is essential to guide clinicians in deciding whether to continue or modify the treatment strategy promptly. This approach may increase the cure rate of DDH, reduce the risk of complications, and minimize physical and psychological distress for affected infants. Despite the well-established association between harness treatment success and deformity severity (Graf classification), observational studies on the impact of perinatal and gestational factors on Pavlik harness outcomes remain limited.

In this study, the clinical outcomes of Pavlik harness treatment were investigated in children with DDH treated at Tianjin Hospital, Tianjin, between 2013 and 2023. Perinatal and gestational factors potentially influencing treatment results were analyzed, aiming to lay the groundwork for improved assessment of Pavlik harness treatment outcomes. Moreover, extending beyond conventional imaging-based severity stratification, the present study integrated perinatal and gestational exposure factors. Our results indicate that specific perinatal factors may differentially influence the effectiveness of Pavlik harness therapy among children with distinct Graf classifications, offering a clinical foundation for early risk stratification and personalized surveillance.

## 2 Materials and methods

### 2.1 Participants

All children who screened positive or were suspected positive for DDH at district-level and municipal-level women and children's health centers in Tianjin between January 2013 and December 2023 and were subsequently referred to Tianjin Hospital for Pavlik harness treatment were enrolled as the study subjects. Eligibility criteria were defined as follows: diagnosis of DDH confirmed at Tianjin Hospital; ultrasound Graf classification (11) of type IIc or above; age ≤ 6 months at the time of treatment initiation; treatment with the Pavlik harness; availability of complete ultrasound imaging data; and informed consent provided by the guardians with voluntary participation in this study. The following exclusion criteria were applied: neuromuscular disorders, syndromic hip dislocation, previous treatment for DDH, use of non-Pavlik harness treatment modalities, incomplete essential data, and failure to achieve the outcome assessment time point. This study was conducted as a retrospective cohort study using data extracted from a prospectively maintained institutional database. Informed consent was obtained from the parents or legal guardians of all participating infants at the time of treatment, permitting the use of their clinical data for future research. The study protocol was approved by the Ethics Committee of Tianjin Center for Women and Children's Health (ethics approval number: WJWKJ-02), which waived the requirement for additional consent given the retrospective design and use of anonymized data. A flowchart of the recruitment process is shown in Figure 1. For patients with bilateral DDH, the unit of analysis in this study was the patient rather than the individual hip. This is because perinatal and gestational factors-the primary exposures of interest-are characteristics of the child as a whole and cannot be attributed to a single hip. Accordingly, all statistical analyses were performed at the patient level, and bilateral cases were treated as a single observation per child.

**Figure 1:**
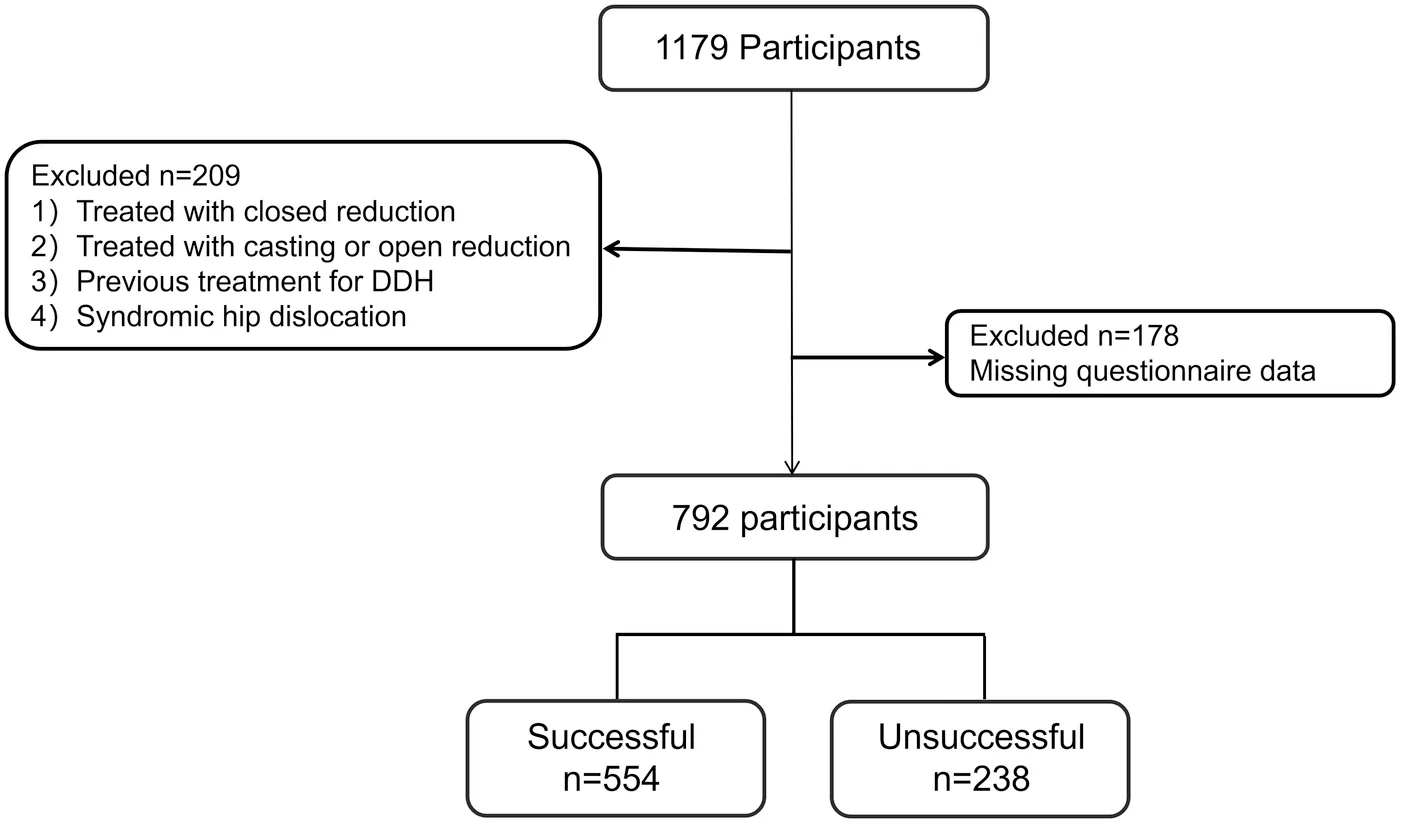
Flow diagram of patient selection and treatment outcomes in this retrospective cohort study of children with DDH treated with the Pavlik harness (2013–2023).

### 2.2 Follow-up protocol and compliance assessment

#### 2.2.1 Follow-up protocol

All patients included in this study were managed according to a standardized institutional protocol for Pavlik harness treatment (Method S1). Ultrasound examinations were performed using the Graf method at the following predefined intervals:

**2 weeks after initiation:** to confirm initial reduction and adjust harness fit if necessary.
**6 weeks:** to assess early progress and detect any loss of reduction.
**12 weeks:** to determine treatment outcome (success or failure).
**Every 4–6 weeks thereafter:** until treatment completion or failure determination.

Treatment success was defined as achievement of Graf type Ia or Ib on ultrasonographic examination (indicating a well-centered femoral head with adequate acetabular coverage) at the 12-week assessment, maintained throughout the follow-up period. Treatment failure was defined as: (1) failure to achieve Graf type Ia or Ib by 12 weeks; (2) persistence of Graf type IIc or worse (i.e., IIc, D, III, or IV) at the 12-week assessment; (3) loss of reduction after initial successful treatment; (4) progression of dislocation; or (5) development of complications (e.g., femoral head avascular necrosis, skin complications, or harness intolerance) that required discontinuation of Pavlik harness treatment and alternative intervention.

#### 2.2.2 Compliance assessment

At the time of Pavlik harness initiation, all families received standardized education regarding harness use, including instructions on daily wear time (at least 23 h per day), proper harness adjustment, skin care, and follow-up schedules, delivered by trained pediatric orthopedic nurses. At each follow-up visit, the treating physician assessed harness fit and queried families regarding wear time and any difficulties encountered. Families reporting suboptimal adherence received additional counseling and reinforcement. However, we acknowledge that objective compliance data (e.g., sensor-based monitoring) were not systematically collected in this study.

### 2.3 Methods

#### 2.3.1 Questionnaire surve

Baseline characteristics of children with DDH treated with the Pavlik harness and maternal perinatal and gestational information were retrospectively collected via a questionnaire. Baseline characteristics of the children included sex, Graf classification, gestational age at birth, and birth weight. Maternal perinatal and gestational information included: paternal and maternal education levels, maternal parity, amniotic fluid status, intrauterine position, mode of delivery, infant feeding mode, gravidity, parity, presence of nocturnal leg cramps during the second and third trimesters, maternal supplementation with vitamin AD preparations during pregnancy, maternal milk consumption during pregnancy, and maternal calcium supplementation during pregnancy.

#### 2.3.2 Evaluation criteria for treatment outcomes

Treatment success was defined as restoration of ultrasound Graf classification from type IIc to type Ia or Ib, accompanied by stable hip joint stability following Pavlik harness treatment; otherwise, treatment was considered unsuccessful. Outcome assessments were confirmed by at least one pediatric orthopedic attending physician and at least one pediatric hip ultrasonographer. When a disagreement arose between the two assessors, a third assessor—another pediatric orthopedic chief physician—was randomly selected to re-evaluate the outcome. The final result was then confirmed by consensus among all involved assessors.

#### 2.3.3 Definitions of relevant influencing factors

Gestational age at birth was categorized according to standard criteria: preterm (<37 weeks), term (37–42 weeks), and post-term (>42 weeks) (12, 13). Birth weight was categorized as low birth weight (<2,500 g), normal birth weight (2,500–3,999 g), and macrosomia (≥4,000 g) (12, 13). Amniotic fluid status was classified according to the deepest vertical pocket (DVP) as normal (2–8 cm) or abnormal (<2 cm or >8 cm). Intrauterine position was defined as cephalic or non-cephalic based on the fetal presentation prior to delivery. Maternal milk consumption during pregnancy was classified as: none (0 times/week), occasional (1–2 times/week), and regular (≥3 times/week).

### 2.4 Statistical analysis

Statistical analysis was performed using SPSS 24.0 (SPSS, Chicago, IL, USA). Continuous variables were compared using the independent samples *t*-test (for normally distributed data) or the Mann–Whitney *U* test (for non-normally distributed data). Categorical variables were analyzed using the Chi-square test (or Fisher's exact test when appropriate). Ordinal variables were compared using the Kruskal–Wallis H test. All comparisons were performed between independent groups. Normality was assessed using the Shapiro–Wilk test.

Logistic regression analysis was performed to test the association between perinatal and gestational factors associated with Pavlik Harness outcomes in DDH, estimating each factor's odds ratio (OR) and 95% confidence interval (CI). A logistic regression model incorporating the selected influencing factors was developed. For multivariable analysis, candidate variables were screened using univariate logistic regression, with *P* < 0.10 as the entry criterion. Variables of established clinical importance based on previous literature were also retained regardless of their univariate *p*-value. Multivariable binary logistic regression was then performed using forward stepwise selection based on the likelihood ratio test, and backward elimination was conducted to confirm the robustness of the final model. The final model was selected based on the best balance between goodness-of-fit and parsimony, as assessed by the Hosmer-Lemeshow test. Multicollinearity was assessed using the variance inflation factor (VIF), with a threshold of 5 indicating significant collinearity. Patients with missing data for any of the variables under investigation were excluded from the study; therefore, the final analytical dataset contained no missing values, and no imputation was performed.

The predicted probability of successful treatment was calculated for each participant. To evaluate the model's discriminative capacity, a receiver operating characteristic (ROC) curve was constructed, with the predicted probability serving as the test variable and the observed treatment outcome as the state variable. The area under the ROC curve (AUC) was computed, and an AUC exceeding 0.7 was considered acceptable. To assess the internal validity of the prediction model and to correct for overfitting optimism, bootstrap internal validation with 200 resampling iterations was performed. The optimism-corrected C-index (concordance index) was calculated to provide a more unbiased estimate of the model's discriminative ability. A nomogram was then created to predict failure Pavlik harness treatment risk based on perinatal and gestational factors. Statistical significance was set at *P* < 0.05 for all hypotheses.

## 3 Results

### 3.1 Baseline characteristics

This study included a total of 792 children (910 hips) with DDH who met the inclusion criteria and received treatment with the Pavlik harness. For children with bilateral DDH (*n* = 118), the more severely affected hip was used to assign a single Graf classification at the patient level. This approach is consistent with the patient-level nature of the primary exposures and ensures that each child contributed one observation to the analysis. The mean age at treatment initiation was 70.5 ± 35.0 days. Among these, successful treatment was achieved in 554 children (637 hips), while 238 children (273 hips) experienced treatment failure, resulting in a treatment success rate of 69.95%. By sex, the cohort comprised 76 boys (87 hips) and 716 girls (823 hips).

### 3.2 Comparison of perinatal and gestational factors between patients with successful and unsuccessful Pavlik harness treatment for DDH

Baseline characteristics of children with DDH treated with the Pavlik harness and maternal perinatal and gestational information were shown in Table 1. The treatment success rate of the Pavlik harness for DDH showed statistically significant differences in relation to the factors including: Graf classification, maternal education level, paternal education level, intrauterine position, parity, maternal combined vitamin A and D supplementation during pregnancy, maternal milk consumption during pregnancy and maternal calcium supplementation during pregnancy (all *P* ＜ 0.05).

**Table 1:** Comparison of baseline characteristics between children with successful and unsuccessful Pavlik harness treatment for DDH, and univariate screening of candidate predictors (*n* = 792).

Factors	*n*	Successful(*n* = 554)	Unsuccessful(*n* = 238)	*χ* ^2^	*p* value
Gender				0.324	0.569
Female	716	503 (70.3)	213 (29.7)		
Male	76	51 (67.1)	25 (32.9)		
Graf classification				60.248^a^	<0.001
IIc	306	245 (80.1)	61 (19.9)		
D or III	385	280 (72.7)	105 (27.3)		
IV	101	29 (28.7)	72 (71.3)		
Maternal education levels				14.834^a^	<0.001
Middle school or below	237	142 (59.9)	95 (40.1)		
High school or technical secondary school	164	117 (71.3)	47 (28.7)		
Associate degree	162	121 (74.7)	41 (25.3)		
Bachelor's degree or above	229	174 (76.0)	55 (24.0)		
Paternal education levels				12.520^a^	<0.001
Middle school or below	257	157 (61.1)	100 (38.9)		
High school or technical secondary school	132	94 (71.2)	38 (28.8)		
Associate degree	178	135 (75.8)	43 (24.2)		
Bachelor's degree or above	225	168 (74.7)	57 (25.3)		
Gestational age at birth				3.467^a^	0.063
<37 weeks	37	28 (75.7)	9 (24.3)		
37–42 weeks	719	506 (70.4)	213 (29.6)		
>42 weeks	36	20 (55.6)	16 (44.4)		
Birth weight				0.070^a^	0.791
<2,500 g	43	28 (65.1)	15 (34.9)		
2,500–3,999 g	479	337 (70.4)	142 (29.6)		
≥4,000 g	270	189 (70.0)	81 (30.0)		
Type of pregnancy				0.929	0.335
Singleton pregnancy	769	540 (70.2)	229 (29.8)		
Multiple pregnancy	23	14 (60.9)	9 (39.1)		
Amniotic fluid status				1.699	0.192
Normal	686	475 (69.2)	211 (30.8)		
Abnormal	106	79 (74.5)	27 (25.5)		
Intrauterine position				19.768	<0.001
Cephalic	671	490 (73.0)	181 (27.0)		
Non-cephalic	121	64 (52.9)	57 (47.1)		
Mode of delivery				0.502	0.478
Vaginal delivery	285	196 (68.8)	89 (31.2)		
Cesarean section	507	358 (70.6)	149 (29.4)		
Infant feeding mode				4.104	0.128
Breastfeeding	529	358 (67.7)	171 (32.3)		
Formula feeding	215	159 (74.0)	56 (26.0)		
Mixed feeding	48	37 (77.1)	11 (22.9)		
Gravidity				0.059^a^	0.808
1	492	343 (69.7)	149 (30.3)		
2	175	122 (69.7)	53 (30.3)		
≥3	125	89 (71.2)	36 (28.8)		
Parity				12.466	<0.001
1	612	409 (66.8)	203 (33.2)		
≥2	180	145 (80.6)	35 (19.4)		
Presence of nocturnal leg cramps during the second and third trimesters				0.427	0.514
No	452	312 (69.0)	140 (31.0)		
Yes	340	242 (71.2)	98 (28.8)		
Maternal combined vitamin A and D supplementation during pregnancy				20.390	<0.001
No	474	303 (63.9)	171 (36.1)		
Yes	318	251 (78.9)	67 (21.1)		
Maternal milk consumption during pregnancy				19.114^a^	<0.001
None	116	66 (56.9)	50 (43.1)		
Occasional	377	255 (67.6)	122 (32.4)		
Regular	299	233 (77.9)	66 (22.1)		
Maternal calcium supplementation during pregnancy				9.055	0.003
No	262	165(63.0)	97(37.0)		
Yes	530	389(73.4)	141(26.6)		

a Calculated using the Kruskal–Wallis *H* test. All data are reported at the patient level (*n* = 792). For children with bilateral DDH, the more severely affected hip was used to assign a single Graf classification, and the outcome was defined at the child level based on the worse-performing hip. Perinatal and gestational factors are child-level exposures.

### 3.3 Associations between perinatal and gestational factors and treatment outcomes of the Pavlik harness for DDH

Logistic regression model was used to assess the association perinatal and gestational factors and treatment outcomes of the Pavlik Harness for DDH, as shown in Table 2. The results showed that low Graf classification (OR_1_ = 7.081, 95%*CI* *=* 4.215–11.894; OR_2_ = 9.833, 95%*CI* = 5.694–16.979), maternal education level of college or above (OR_1_ = 1.963, 95%*CI* = 1.209–3.187; OR_2_ = 2.200，95%*CI* = 1.338–3.616; OR_3_ = 2.332, 95%*CI* = 1.480–3.677), parity > 1 (OR = 2.447, 95% *CI* = 1.548–3.870), maternal combined vitamin A and D supplementation during pregnancy, (OR = 1.875, 95% *CI* = 1.301–2.702) and regular maternal milk consumption during pregnancy (OR = 2.435, 95% *CI* = 1.453–4.079) were significantly associated with higher success rate of Pavlik harness therapy. Conversely, non-cephalic intrauterine position (OR = 0.356, 95% CI = 0.227–0.558) was significantly associated with a lower success rate. While other perinatal and gestational factors had no significant associations with treatment outcomes of the Pavlik Harness for DDH.

**Table 2:** Multivariable logistic regression analysis of perinatal and gestational factors associated with Pavlik harness treatment success in children with DDH (*n* = 792).

Variable	*B*	*SE*	Wald *χ*^2^	*P* value	*OR*(*95%CI*)
Graf classification (Ref: Type IV)			70.935	0.000	
Type D or III	1.957	0.265	54.711	0.000	7.081 (4.215–11.894)
Type IIc	2.286	0.279	67.250	0.000	9.833 (5.694–16.979)
Maternal education level (Ref: Middle school or below)			17.224	0.009	
High school/Technical secondary school	0.675	0.247	7.450	0.006	1.963 (1.209–3.187)
Associate degree	0.788	0.254	9.663	0.002	2.200 (1.338–3.616)
Bachelor's degree or above	0.847	0.232	13.308	0.000	2.332 (1.480–3.677)
Paternal education level (Ref: Middle school or below)			5.570	0.135	
High school/Technical secondary school	0.445	0.280	2.580	0.112	1.560 (0.901–2.700)
Associate degree	0.559	0.265	1.829	0.035	1.750 (1.041–2.942)
Bachelor's degree or above	0.470	0.272	1.844	0.084	1.599 (0.938–2.726)
Intrauterine position (Ref: Cephalic)	−1.033	0.229	20.359	0.000	0.356 (0.227–0.558)
Parity (Ref: 1)	0.895	0.234	14.648	0.000	2.447 (1.548–3.870)
Maternal combined vitamin A and D supplementation during pregnancy (Ref: No)	0.629	0.186	11.380	0.001	1.875 (1.301–2.702)
Maternal milk consumption during pregnancy (Ref: None)			12.026	0.002	
Occasional (1–2 times/week)	0.444	0.245	3.280	0.070	1.560 (0.964–2.523)
Regular (≥3 times/week)	0.890	0.263	11.425	0.001	2.435 (1.453–4.079)
Maternal calcium supplementation during pregnancy (Ref: No)	0.227	0.186	1.487	0.223	1.255 (0.871–1.809)

OR, odds ratio; CI, confidence interval; Ref, reference category. The outcome variable was treatment success (successful vs. unsuccessful). Variables with *P* < 0.05 in the multivariate analysis were considered statistically significant. The overall model fit was assessed using the Hosmer-Lemeshow test (*χ*^2^ = 5.984*, P* = 0.584).

### 3.4 Interaction analysis between Graf classification and perinatal/gestational factors

To investigate whether the severity of DDH, as defined by Graf classification, interacts with perinatal and gestational factors influencing the outcome of Pavlik harness treatment, interaction terms were constructed between each of the five perinatal/gestational factors that showed statistical significance in logistic regression analysis (maternal education level, intrauterine position, parity, maternal combined vitamin A and D supplementation during pregnancy, and regular maternal milk consumption) and the Graf classification. These five interaction terms were then individually added to the main-effects logistic regression model described above, and their significance was assessed.

As shown in Table 3, significant interaction effects were observed between parity and Graf classification (*P* *<* 0.05), as well as between regular maternal milk consumption and Graf classification (*P* *<* 0.05). No significant interactions were detected for the other three factors (all *P* > 0.05).

**Table 3:** Interaction analysis between Graf classification and perinatal/gestational factors for Pavlik harness treatment success in children with DDH.

Interaction terms	*B*	*SE*	Wald *χ*^2^value	*P* value
Graf classification * Maternal education level	0.002	0.112	0.000	0.984
Graf classification * Intrauterine position	−0.142	0.394	0.131	0.717
Graf classification * Parity	0.749	0.374	4.020	0.045
Graf classification * maternal combined vitamin A and D supplementation during pregnancy	0.022	0.280	0.006	0.939
Graf classification * Maternal milk consumption during pregnancy	0.239	0.068	12.332	0.000

### 3.5 Stratified analysis by Graf classification

Stratified logistic regression analysis was performed according to Graf classification, with a total of three strata. Stratum 1 consisted of 306 patients with Graf type IIc, Stratum 2 comprised 385 patients with Graf type D or III, and Stratum 3 included 101 patients with Graf type IV.

As shown in Table 4, in stratum 1, parity > 1 (adjusted OR = 3.735, 95% CI = 1.530–9.115) was significantly associated with higher success rate of Pavlik harness therapy in patients with type IIc. In stratum 2, parity > 1 (adjusted OR = 2.211, 95% CI = 1.183–4.130) and regular maternal milk consumption during pregnancy (adjusted OR = 3.315, 95% CI = 1.650–6.661) were significantly associated with higher success rate of Pavlik harness therapy in patients with type D or III. There was no significantly associated between perinatal/gestational factors and treatment outcomes of the Pavlik Harness for patients with type IV.

**Table 4:** Stratified logistic regression analysis of perinatal/gestational factors associated with pavlik harness treatment success by Graf classification (stratum 1: Graf type IIc; stratum 2: Graf types D/III; stratum 3: Graf type IV).

Graf classification	Perinatal/gestational factors with interaction effect	*P* value	Adjusted OR (95% CI)
Type IIc (Stratified 1)	Parity (Ref: 1)	0.003	3.735 (1.530–9.115)
	Maternal milk consumption during pregnancy (Ref: None)	0.105	
	Occasional (1–2 times/week)	0.520	1.294 (0.590–2.839)
	Regular (≥3 times/week)	0.055	2.304 (0.982–5.407)
Type D or III (Stratified 2)	Parity (Ref: 1)	0.013	2.211 (1.183–4.130)
	Maternal milk consumption during pregnancy (Ref: None)	0.003	
	Occasional (1–2 times/week)	0.058	1.867 (0.979–3.560)
	Regular (≥3 times/week)	0.001	3.315 (1.650–6.661)
Type IV (Stratified 3)	Parity (Ref: 1)	0.657	0.768 (0.240–2.460)
	Maternal milk consumption during pregnancy (Ref: None)	0.147	
	Occasional (1–2 times/week)	0.153	3.200 (0.649–15.775)
	Regular (≥3 times/week)	0.054	4.952 (0.976–25.140)

### 3.6 Receiver operating curve (ROC) analysis

Perinatal/gestational factors identified through Logistic regression model were analyzed in the ROC model to assess their diagnostic value. As shown in Figure 2A, in the overall prediction model, among all perinatal/gestational factors significantly associated with Pavlik harness treatment outcomes, the Graf classification alone had the highest area under the curve (AUC) at 0.658. The combination of Graf classification, parity, intrauterine position and maternal milk consumption during pregnancy yielded an AUC of 0.723, and the inclusion of all six perinatal/gestational factors (graf classification, maternal education level, intrauterine position, parity, maternal combined vitamin A and D supplementation during pregnancy, and maternal milk consumption during pregnancy) resulted in an AUC of 0.796. After internal validation using bootstrap resampling with 200 iterations, the optimism-corrected C-index was 0.781 (Supplementary Figure S1A), indicating that the model maintained good discriminative ability after accounting for overfitting.

**Figure 2:**
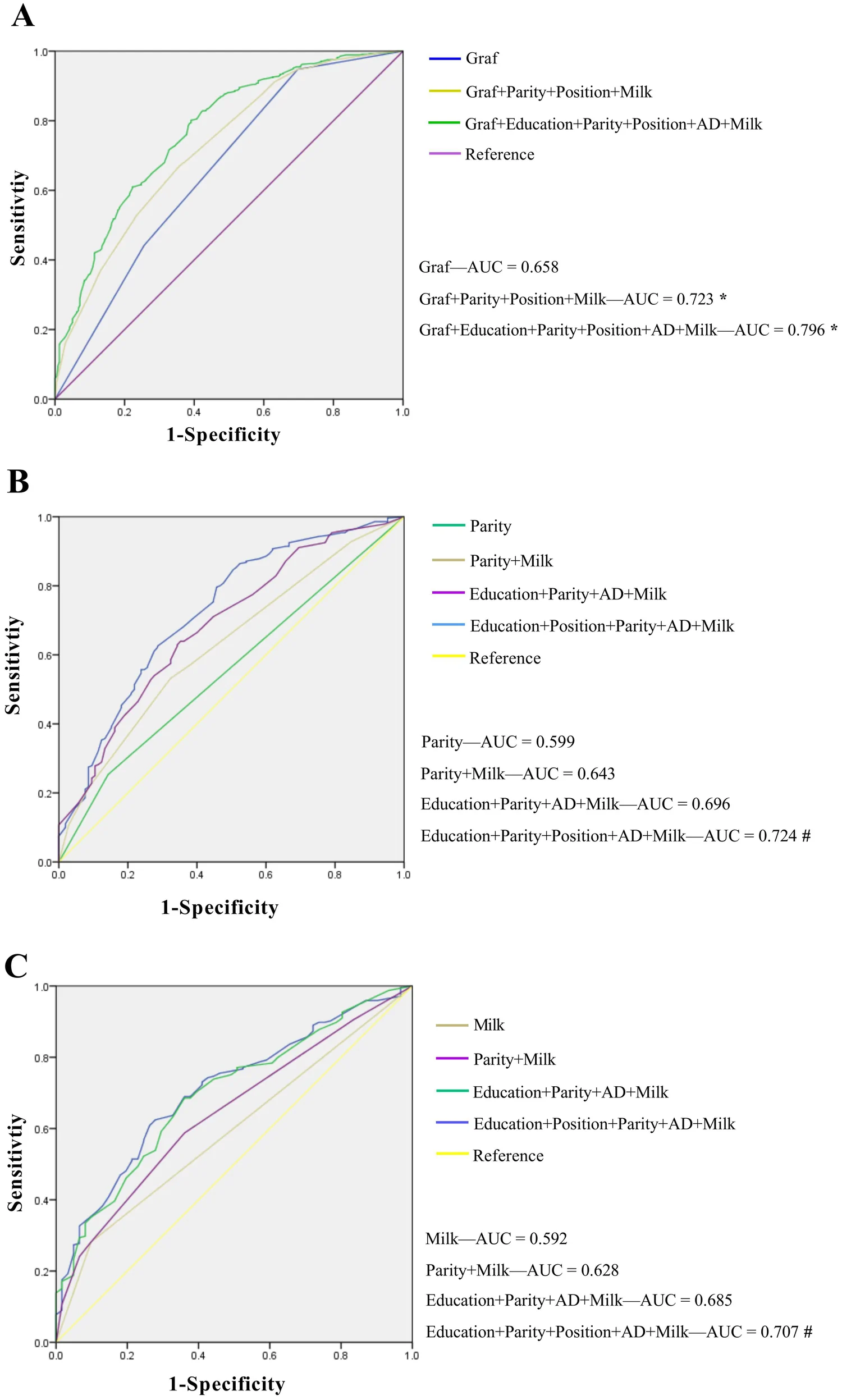
Receiver operating characteristic (ROC) curves for the overall and graf-stratified prediction models of Pavlik harness treatment success in DDH. **(A)** Overall model incorporating Graf classification and five perinatal/gestational factors (AUC = 0.796; bootstrap-corrected C-index = 0.781). **(B)** Stratum 1 (Graf type IIc) model incorporating five perinatal/gestational factors (AUC = 0.724; bootstrap-corrected C-index = 0.719). **(C)** Stratum 2 (Graf types D/III) model incorporating five perinatal/gestational factors (AUC = 0.707; bootstrap-corrected C-index = 0.705). *Indicates the AUC values significantly different from Graf classification. #Indicates the AUC values significantly different from Parity. &Indicates the AUC values significantly different from Maternal milk consumption during pregnancy. *AUC* area under the curve; *Graf* Graf classification; *Milk* maternal milk consumption during pregnancy; *Education* maternal education level; *AD* maternal combined vitamin A and D supplementation during pregnancy.

In the prediction model stratified by Graf classification, as shown in Figure 2B, the ROC curves of Stratum 1 showed that parity had the highest area under the curve (AUC) at 0.599. The combination of parity and maternal milk consumption during pregnancy resulted in an AUC of 0.643, and the combination of maternal education level, parity, maternal combined vitamin A and D supplementation during pregnancy and maternal milk consumption during pregnancy yielded an AUC of 0.696. The inclusion of all five perinatal/gestational factors resulted in an AUC of 0.724. After internal validation using bootstrap resampling with 200 iterations, the optimism-corrected C-index was 0.719 (Supplementary Figure S1B). In stratum 2, that maternal milk consumption during pregnancy had the highest area under the curve (AUC) at 0.592. The combination of parity and maternal milk consumption during pregnancy yielded an an AUC of 0.628, and the combination of maternal education level, parity, maternal combined vitamin A and D supplementation during pregnancy and maternal milk consumption during pregnancy resulted in an AUC of 0.685. The inclusion of all five perinatal/gestational factors resulted in an AUC of 0.707. These results were shown in Figure 2C. Bootstrap internal validation with 200 resampling iterations yielded an optimism-corrected C-index of 0.705 (Supplementary Figure S1C). However, within the stratum 3, the predictive performance of the model was limited, with a C-index of 0.675 and yielded an optimism-corrected C-index of 0.660 (Supplementary Figure S2).

### 3.7 Nomogram analysis

A nomogram incorporating Graf classification and five perinatal and gestational factors was developed to predict the success rate of Pavlik harness treatment in DDH. The nomogram assigns scores to each factor, with the total score corresponding to the predicted probability of treatment success. The model demonstrated excellent discrimination, with a C-index of 0.796, indicating strong predictive capability (Figure 3, Supplementary Table S5).

**Figure 3:**
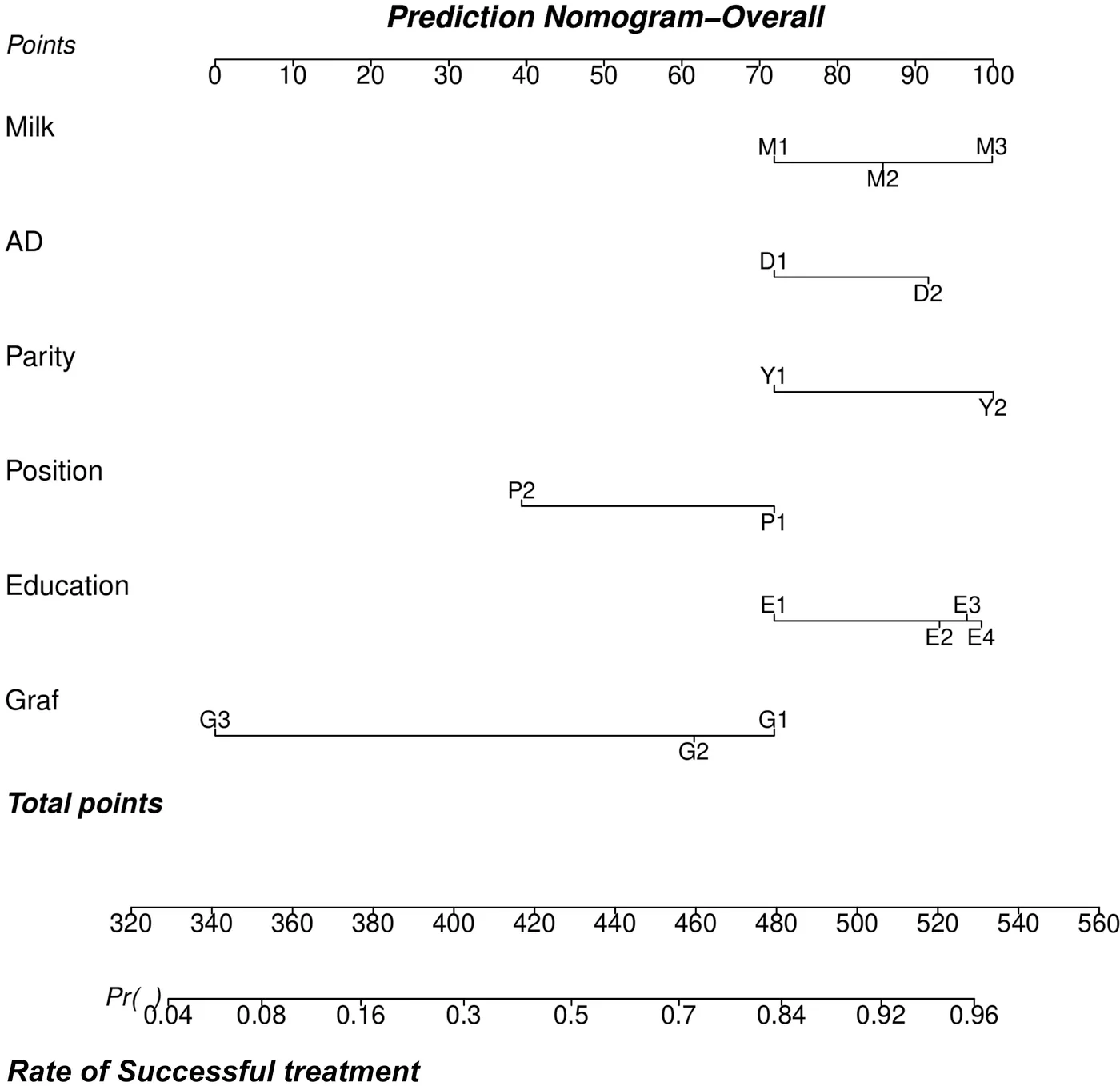
Nomogram for predicting the probability of Pavlik harness treatment success in DDH based on Graf classification and five perinatal/gestational factors (overall model). The nomogram incorporates six variables: maternal milk consumption during pregnancy (M), maternal combined vitamin A and D supplementation during pregnancy (D), parity (Y), intrauterine position (P), maternal education level (E), and Graf classification (G) To use the nomogram, the score corresponding to each patient's variable category is identified on the “Points” scale. The sum of these scores is located on the “Total Points” scale, and a vertical line is drawn downward to the “Pr (success)” axis to obtain the predicted probability of treatment success. The model demonstrated acceptable discriminative ability, with a bootstrap-corrected C-index of 0.781 (95% CI: 0.728–0.814). Coding for categorical variables: Milk consumption: M1 = none, M2 = occasional, M3 = regular; Vitamin A and D supplementation: D1 = no, D2 = yes; Parity: Y1 = 1, Y2 = >1; Intrauterine position: P1 = cephalic, P2 = non-cephalic; Maternal education: E1 = middle school or below, E2 = high school/technical secondary school, E3 = associate degree, E4 = bachelor's degree or above; Graf classification: G1 = type IIc, G2 = type D or III, G3 = type IV.

Stratified analyses based on Graf classification were further performed to evaluate the predictive value of the five perinatal and gestational factors using separate nomograms for each stratum. Both prediction models demonstrated acceptable discriminative ability, with C-index of 0.724 (Stratum 1) and 0.707 (Stratum 2), respectively (Figure 4, Supplementary Tables S6, S7). However, we acknowledge that for variables without significant interaction, the stratified coefficients should be interpreted with caution as exploratory findings.

**Figure 4:**
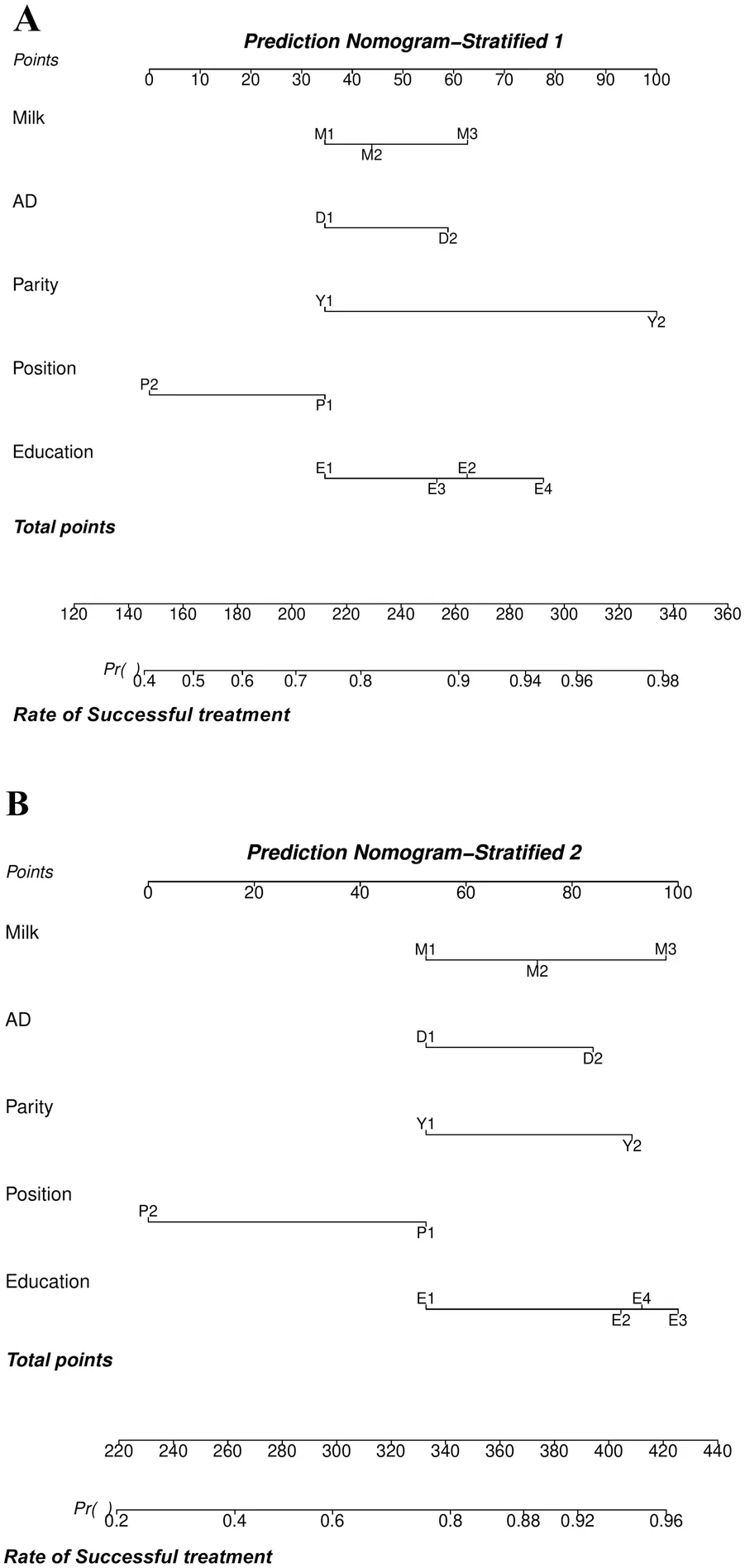
Stratified nomograms for predicting the probability of Pavlik harness treatment success in DDH. **(A)** presents the nomogram for Stratum 1 (Graf type IIc), and **(B)** presents the nomogram for Stratum 2 (Graf types D/III). Each nomogram incorporates five variables: maternal milk consumption during pregnancy (M), maternal combined vitamin A and D supplementation during pregnancy (D), parity (Y), intrauterine position (P), and maternal education level (E) To use each nomogram, the score corresponding to each patient's variable category is identified on the “Points” scale. The sum of these scores is located on the “Total Points” scale, and a vertical line is drawn downward to the “Rate of Successful Treatment” axis to obtain the predicted probability of treatment success. The stratified models demonstrated acceptable discriminative ability, with bootstrap-corrected C-indices of 0.719 for Stratum 1 and 0.705 for Stratum 2. Coding for categorical variables: Milk consumption: M1 = none, M2 = occasional, M3 = regular; Vitamin A and D supplementation: D1 = no, D2 = yes; Parity: Y1 = 1, Y2 = >1; Intrauterine position: P1 = cephalic, P2 = non-cephalic; Maternal education: E1 = middle school or below, E2 = high school/technical secondary school, E3 = associate degree, E4 = bachelor's degree or above. Graf classification was used for stratification and was therefore not included as a predictor in the stratified nomograms.

### 3.8 Model calibration

To assess the calibration of the prediction models, we performed calibration analysis with bootstrap resampling (200 iterations) comparing predicted vs. observed probabilities of treatment success for the overall model and each stratified model. The detailed methods and calibration plots are provided in the supplementary material (Supplementary Figure S3; Supplementary Table S4). The calibration curves for all three models demonstrated good agreement between predicted and observed probabilities, with the curves lying close to the ideal 45° reference line (Supplementary Figure S3). Quantitative calibration metrics are summarized in Supplementary Table S4. The calibration slopes for the overall model, Stratum 1, and Stratum 2 were 0.952 (95% CI: 0.891–1.013), 0.941 (95% CI: 0.872–1.010), and 0.963 (95% CI: 0.905–1.021), respectively, all close to the ideal value of 1.0. The corresponding calibration intercepts were −0.018 (95% CI: −0.082 to 0.046), −0.025 (95% CI: −0.097 to 0.047), and −0.011 (95% CI: −0.079 to 0.057), all close to the ideal value of 0. The Brier scores for the three models were 0.182, 0.191, and 0.175, respectively, well below the non-informative threshold of 0.25. Collectively, these findings indicate no significant systematic over- or under-prediction and confirm acceptable calibration for all.

### 3.9 Decision curve analysis

To evaluate the clinical utility of the prediction models, we performed decision curve analysis (DCA) to quantify the net benefit across a range of threshold probabilities for the overall model and each stratified model. The detailed methods and DCA curves are provided in the Supporting Information (Supplementary Figures S4–S6; Supplementary Table S5). The DCA curves for all three models demonstrated positive net benefit across clinically meaningful threshold ranges, with the model curves lying above both the “Treat All” and “Treat None” reference strategies (Supplementary Figures S4–S6). The net benefit was greatest in the intermediate threshold range (approximately 0.3–0.6 for the overall model), where clinical decision-making is most uncertain. Among the stratified models, the net benefit profile was most favorable in Stratum 1 (Graf type IIc), while Stratum 2 (Graf types D/III) also demonstrated positive net benefit, albeit with somewhat lower magnitude. Quantitative net benefit values at selected thresholds are summarized in Supplementary Table S2. These findings support the potential clinical utility of the nomogram as a decision-support tool for identifying infants at higher risk of Pavlik harness treatment failure.

## 4 Discussion

This study demonstrates that perinatal and gestational factors are significantly associated with the outcomes of Pavlik harness treatment for developmental dysplasia of the hip (DDH). Furthermore, we established an overall prediction model for Pavlik harness treatment outcomes in DDH using logistic regression and ROC analysis. The nomogram incorporating the Graf classification and the five identified perinatal and gestational factors (maternal education level, Intrauterine position, parity, maternal combined vitamin A and D supplementation during pregnancy and maternal milk consumption during pregnancy) effectively facilitates preliminary prediction of Pavlik harness treatment outcomes in infants with DDH. Furthermore, these five factors also provide additional predictive information in the stratified analyses based on Graf classification.

The Pavlik harness represents a primary treatment option for early DDH, with reported success rates exceeding 80% in infants aged less than 6 months at treatment initiation (14, 15). However, the success rate of Pavlik harness treatment tends to be unsatisfactory in DDH infants with Graf type D or above and a positive Barlow sign (16). Additionally, the risk of harness-related complications increases substantially with prolonged treatment duration (17). Therefore, this study investigated perinatal and gestational factors that may influence Pavlik harness treatment outcomes, aiming to advance the prediction window and provide a reference for optimizing treatment strategies for DDH. Our findings indicated that maternal education level was one of the factors influencing the success rate of Pavlik harness treatment. A previous study reported that younger maternal age was associated with a higher risk of DDH, which may be partly attributable to the generally lower educational attainment among younger mothers (18). Compared with fathers, mothers typically assume a more significant role in perinatal care and postnatal parenting. This result may be related to factors such as family health awareness and compliance with follow-up visits. While this association may reflect a genuine biological or socio-economic effect, an alternative interpretation is that maternal education serves as a proxy for treatment adherence. Mothers with higher educational attainment may demonstrate better understanding of treatment rationale, more consistent adherence to the prescribed wear protocol, and more timely follow-up attendance. Previous studies have consistently shown that maternal education and health literacy are strong predictors of adherence across various pediatric conditions (19). Therefore, the observed association between maternal education and treatment outcomes should be interpreted with caution, as it may be confounded by differential adherence. Future studies incorporating objective compliance monitoring are needed to disentangle the independent effects of maternal education from those of treatment adherence.

Additionally, our study identified non-cephalic intrauterine position and primiparity (parity = 1) as factors associated with lower success rates of Pavlik harness treatment. Previous study has indicated that non-cephalic presentation, including breech position, exerts greater mechanical forces on the fetal hip joint, thereby compromising hip stability (20). Another study has also shown that firstborn infants are at an increased risk of DDH (21). This association may be explained by the fact that nulliparous mothers have not experienced prior vaginal delivery, and thus the compressive forces exerted on the fetus during labor are significantly greater than those in multiparous mothers. This mechanism is consistent with the aforementioned theory regarding the influence of intrauterine positioning on hip development.

In this study, maternal combined vitamin A and D supplementation during pregnancy and regular milk consumption during pregnancy were associated with higher success rates of Pavlik harness treatment. During development, the establishment and maturation of the ossification center of the hip joint depend on adequate calcium and vitamin D levels (22). Maternal calcium and vitamin D status may indirectly reflect fetal levels of these nutrients (23). Accumulating evidence has shown that calcium supplementation in the form of dairy products exerts superior effects on bone health compared with non-dairy calcium sources (24, 25). Therefore, special attention should be given to ossification center development and hip joint recovery in DDH infants whose mothers did not supplement with vitamin A and D or had poor milk intake during pregnancy. If the treatment response is unsatisfactory, the therapeutic strategy should be modified promptly to optimize care and achieve early cure. In addition, in this study, the maternal supplementation rate of vitamin A and D during pregnancy was only 40.2%, and the proportion of regular milk consumption during pregnancy was only 37.8%. Therefore, health education during pregnancy should be further strengthened to encourage regular milk intake and appropriate combined vitamin A and D supplementation. However, given the observational nature of this study, these findings should be interpreted with caution. The observed associations may be influenced by unmeasured confounders such as socioeconomic status, health literacy, healthcare access, and treatment adherence. For instance, maternal milk consumption may serve as a proxy for better overall nutritional status or greater health awareness, rather than exerting a direct biological effect on hip development. Similarly, the association between vitamin A and D supplementation and treatment success may reflect better access to prenatal care rather than a specific pharmacological effect. These potential confounders should be carefully considered when interpreting our results.

Our study also identified Graf classification as a significant predictor of Pavlik harness treatment outcomes. Specifically, higher Graf types were associated with lower success rates. This finding is consistent with the results of numerous previous studies (9, 26). Therefore, developing more rational and individualized treatment strategies based on Graf classification (severity) in DDH patients has significant clinical value and practical implications for the management of this condition. In this study, interaction analysis revealed significant interactions between Graf classification and both parity and maternal milk consumption during pregnancy. Further stratified association analysis indicated that, in Stratum 1 (Graf type IIc), parity exhibited the strongest modifying effect among the perinatal and gestational factors. In Stratum 2 (Graf type D or III), both parity and maternal milk consumption during pregnancy contributed notably to the modifying effects. However, in Stratum 3 (Graf type IV), no significant modifying effect was observed for any of the perinatal or gestational factors. In the Graf IV subgroup, where treatment failure rates are known to be substantially higher regardless of other clinical characteristics, the limited performance of our model suggests that disease severity itself is the predominant driver of outcomes. The stratified analyses revealed a clinically important pattern: while the perinatal/gestational factors demonstrated acceptable predictive performance in Graf types IIc and D/III, they had no predictive utility in Graf Type IV hips. This finding suggests that in the most severe DDH category, disease severity itself is such a powerful determinant of treatment failure that additional clinical characteristics-including maternal and perinatal factors-do not materially improve risk stratification. Clinicians should be aware that for Graf Type IV patients, treatment decisions should be guided primarily by disease severity and early response to treatment, rather than by perinatal/gestational factors. The lack of predictive utility in this high-risk subgroup also highlights the need for alternative prognostic markers, such as dynamic ultrasonographic parameters, acetabular index measurements, or early treatment response trajectories, which may provide additional prognostic information beyond static disease severity classification. The overall model's good discriminative ability (C-index = 0.796) appears to be largely driven by the strong association between Graf classification and treatment outcomes, rather than by the independent contributions of perinatal/gestational factors within each stratum. Therefore, while the overall model may be useful for initial risk stratification in the general DDH population, its clinical utility in the most severe cases is limited. Therefore, these findings suggest that the association between perinatal/gestational factors and Pavlik harness treatment outcomes in DDH may vary across different Graf classifications, warranting further validation through prospective studies.

Based on perinatal and gestational factors, we preliminarily constructed both an overall prediction model and Graf classification-stratified prediction models for Pavlik harness treatment outcomes in DDH using logistic regression. ROC and nomogram analyses demonstrated that the overall model exhibited good predictive capability, and the stratified models also showed acceptable performance. The calibration assessment confirmed that both the overall and stratified models were well-calibrated across the range of predicted probabilities, supporting the reliability of the nomogram for clinical risk stratification. The Brier scores for all models were well below the non-informative threshold, further confirming acceptable prediction accuracy. The slightly better calibration observed in the overall model compared with the stratified models may reflect the larger sample size and greater statistical power in the overall cohort. Collectively, all three models demonstrated satisfactory calibration, suggesting that the nomogram provides reliable probability estimates for clinical decision-making across different Graf severity strata.

The DCA confirmed that the nomogram provides a positive net benefit across clinically meaningful threshold ranges for all three models, supporting its potential utility as a decision-support tool in clinical practice. The finding that the net benefit was greatest in the intermediate threshold range (approximately 0.3–0.6) is particularly relevant, as this is the range where clinical uncertainty is highest and decision support is most needed. For very low-risk or very high-risk patients, clinical judgment alone may be sufficient, and the incremental benefit of the nomogram is limited. However, for patients with intermediate-risk profiles, the nomogram may help clinicians identify those who require more intensive monitoring or earlier alternative interventions. The observation that the net benefit was most favorable in Stratum 1 (Graf type IIc) and somewhat lower in Stratum 2 (Graf types D/III) is consistent with the clinical expectation that prediction models may have greater utility in milder disease categories, where treatment decisions are more variable and prognostic uncertainty is higher. In the more severe categories, disease severity itself is often the dominant driver of treatment decisions, potentially reducing the incremental value of additional predictors. The Graf-stratified nomograms were designed to provide clinicians with stratum-specific prediction tools for patients within each Graf grade, reflecting clinical practice where management decisions are inherently stratified by disease severity. However, we acknowledge that the statistical justification for stratification is stronger for variables with significant interaction effects (parity and milk consumption) than for those without (maternal education, intrauterine position, vitamin A and D supplementation). For the latter, the stratified coefficients should be interpreted as exploratory and hypothesis-generating, and the overall nomogram remains the primary prediction tool. Future studies with larger sample sizes within each stratum are warranted to validate these exploratory findings. Nonetheless, these findings may serve as a stratified reference to support early monitoring and individualized clinical decision-making in the management of DDH patients undergoing Pavlik harness treatment.

Our study has several strengths. First, our approach was not limited to evaluating disease severity or imaging features alone. Instead, we integrated perinatal and gestational factors with Graf classification, establishing a new paradigm for risk identification in DDH that captures both disease-specific and patient-level determinants of treatment outcomes. This multidimensional framework moves beyond traditional single-factor analyses by recognizing that Pavlik harness outcomes are shaped by the interplay between baseline hip morphology and maternal-perinatal characteristics. Second, the stratified analysis revealed differential effects of perinatal factors across Graf types, which may facilitate more individualized treatment decisions. Specifically, our finding that certain perinatal factors—particularly parity and maternal milk consumption—demonstrated significant interactions with Graf classification underscores the value of tailored risk assessment based on disease severity. Third, the selected perinatal and gestational factors are readily available in routine clinical practice through standard obstetric history-taking, enhancing the feasibility and generalizability of our findings across diverse clinical settings without requiring additional specialized testing. Fourth, the development of both overall and Graf-stratified nomograms provides clinicians with flexible tools that can be applied either broadly or in a severity-specific manner, depending on the clinical context. Fifth, we performed comprehensive model evaluation—including discrimination (ROC/AUC), calibration (calibration plots, calibration slope/intercept, Brier score), internal validation (bootstrap resampling), and decision curve analysis—which collectively confirm the robustness and clinical utility of our prediction models. Finally, the large sample size (792 children, 910 hips) and the relatively long study period (2013–2023) provide sufficient statistical power to detect meaningful associations and enhance the generalizability of our findings to similar clinical populations.

This study also has several limitations that need to be acknowledged. First, as a single-center retrospective study, we are unable to establish causal relationships, and the findings lack external validation. Second, the assessment of perinatal and gestational factors was relatively crude; for instance, maternal milk consumption was not quantified, and the dosage and formulation of calcium and vitamin A and D supplements were not specified. Third, although we adjusted for Graf classification in the multivariable model and performed stratified analyses to control for disease severity, the limited predictive performance in the Graf IV subgroup warrants caution when applying the model to the most severe DDH cases. The restricted outcome variability and relatively small sample size in this stratum may have reduced the statistical power to detect additional predictive signals. Future studies with larger cohorts of Graf IV patients are needed to further validate and refine the model for this high-risk subgroup. Fourth, two of the five predictors-maternal milk consumption and vitamin A and D supplementation during pregnancy-were collected retrospectively via questionnaire over a ten-year period (2013–2023). This extended timeframe raises concerns about recall bias, particularly for participants enrolled early in the study period. Although the use of broad categorical response options may have partially mitigated this bias, residual misclassification cannot be excluded. Future prospective studies with real-time dietary recording or biomarker-based assessment are warranted to validate these findings. Fifth, a number of potentially eligible patients were excluded from the analysis due to incomplete questionnaire data. While a comparison of key baseline characteristics between included and excluded patients revealed no significant differences, we cannot exclude the possibility of selection bias if patients with missing data differed systematically in unmeasured characteristics. The generalizability of our findings should be interpreted with this limitation in mind. Finally, the absence of biochemical and imaging biomarkers limited our ability to provide more objective and direct evaluations of treatment outcomes. Future studies with refined protocols and improved experimental designs are warranted to provide more detailed data supporting the evaluation of reduction outcomes following Pavlik harness treatment.

## 5 Conclusion

In this retrospective cohort study, we identified five perinatal and gestational factors (maternal education level, intrauterine position, parity, maternal combined vitamin A and D supplementation during pregnancy and maternal milk consumption during pregnancy) independently associated with Pavlik harness treatment success in DDH. The overall nomogram demonstrated good discriminative ability, with acceptable performance in Graf types IIc and D/III; however, it showed no predictive value in type IV hips. Calibration and decision curve analyses supported the model's clinical utility for intermediate-risk patients. Given the observational design, these findings should be interpreted as exploratory associations rather than causal determinants, and external validation is warranted before clinical implementation.

## Data Availability

The original contributions presented in the study are included in the article/Supplementary Material, further inquiries can be directed to the corresponding author/s.
