# Supplementary material for: Perinatal and gestational factors associated with Pavlik harness outcomes in developmental dysplasia of the hip: a retrospective cohort study
Source: Front Pediatr. 2026 Sep 28;14:1938073. doi: 10.3389/fped.2026.1938073 (PMC13654962; doi:10.3389/fped.2026.1938073)
Supplement: Supplementary file 1 [file Supplementaryfile1.doc]

**Method 1**.

Treatment protocol

All patients included in this study were treated according to a standardized institutional protocol for Pavlik harness application and management at Tianjin Hospital. The harness used was the same model for all patients.

Harness application. The Pavlik harness was applied by a standardized clinical team consisting of three senior pediatric orthopedic surgeons at Tianjin Hospital, each with over 5 years of experience in DDH treatment. The harness was positioned with the hips flexed at 90-100° and abducted to 40-60°, with the goal of achieving a stable, concentric reduction. All initial applications were performed under ultrasound guidance to confirm femoral head reduction, with the harness adjusted until the femoral head was well-centered in the acetabulum.

Follow-up adjustments. Harness adjustments were made at each scheduled follow-up visit (at 2, 6, and 12 weeks) based on ultrasonographic findings and clinical stability, with the goal of maintaining a stable reduction while gradually reducing the degree of abduction as the hip matured. All adjustments were performed by the same clinical team using the standardized protocol.

Treatment discontinuation. Pavlik harness treatment was discontinued when: (1) a stable reduction with α angle ≥ 60° (Graf type Ia or Ib) was maintained for at least 3 consecutive months; (2) treatment failure was determined at the 12-week assessment (persistent dislocation or instability, Graf type IIc or worse); or (3) complications (e.g., harness intolerance, skin complications, or femoral head avascular necrosis) occurred that required alternative treatment.

**Method 2**.

Sample size

The minimum required sample size was determined based on the events per variable (EPV) rule for logistic regression model development. With six candidate predictors (Graf classification, maternal education level, parity, intrauterine position, vitamin AD supplementation, and milk consumption) and an estimated treatment success rate of approximately 70% (554 successes out of 792 cases), an EPV of 10 would require at least 100 patients (10 × 6 / 0.70), an EPV of 20 would require at least 171 patients (20 × 6 / 0.70), and an EPV of 50 would require at least 429 patients (50 × 6 / 0.70). Our final cohort of 792 patients (554 successes, 238 failures) yielded an EPV of approximately 92 (554 successes / 6 predictors), which exceeds all recommended thresholds and provides adequate statistical power to detect meaningful associations and to develop a stable prediction model without significant overfitting.

Table 1. Multicollinearity assessment among predictors in overallpredictive model

| **Variable** | **VIF** | **Tolerance** |
| --- | --- | --- |
| Graf classification | 1.020 | 0.980 |
| Maternal education levels | 1.046 | 0.956 |
| Parity | 1.010 | 0.990 |
| Intrauterine position | 1.025 | 0.976 |
| Maternal supplementation with vitamin AD preparations during pregnancy | 1.028 | 0.973 |
| Maternal milk consumption during pregnancy | 1.038 | 0.963 |

VIF, variance inflation factor; Tolerance = 1/VIF. All VIF values were < 2, indicating no significant multicollinearity.

Table 2. Multicollinearity assessment among predictors in stratum 1

| **Variable** | **VIF** | **Tolerance** |
| --- | --- | --- |
| Maternal education levels | 1.055 | 0.948 |
| Parity | 1.014 | 0.986 |
| Intrauterine position | 1.023 | 0.978 |
| Maternal supplementation with vitamin AD preparations during pregnancy | 1.036 | 0.965 |
| Maternal milk consumption during pregnancy | 1.049 | 0.953 |

VIF, variance inflation factor; Tolerance = 1/VIF. All VIF values were < 2, indicating no significant multicollinearity.

Table 3. Multicollinearity assessment among predictors in stratum 2

| **Variable** | **VIF** | **Tolerance** |
| --- | --- | --- |
| Maternal education levels | 1.028 | 0.973 |
| Parity | 1.006 | 0.994 |
| Intrauterine position | 1.027 | 0.974 |
| Maternal supplementation with vitamin AD preparations during pregnancy | 1.027 | 0.973 |
| Maternal milk consumption during pregnancy | 1.034 | 0.967 |

VIF, variance inflation factor; Tolerance = 1/VIF. All VIF values were < 2, indicating no significant multicollinearity.

Table 4. Multicollinearity assessment among predictors in stratum 3

| **Variable** | **VIF** | **Tolerance** |
| --- | --- | --- |
| Maternal education levels | 1.144 | 0.874 |
| Parity | 1.020 | 0.980 |
| Intrauterine position | 1.063 | 0.940 |
| Maternal supplementation with vitamin AD preparations during pregnancy | 1.016 | 0.985 |
| Maternal milk consumption during pregnancy | 1.128 | 0.887 |

VIF, variance inflation factor; Tolerance = 1/VIF. All VIF values were < 2, indicating no significant multicollinearity.

**Figure 1** Bootstrap-corrected ROC curves for the prediction model. (A) presents the bootstrap-corrected ROC curves for overall prediction model (B) presents the bootstrap-corrected ROC curves for Stratum 1, and (C) presents the curves for Stratum 2. The solid blue line represents the full model including all five perinatal/gestational factors (maternal education, intrauterine position, parity, vitamin AD supplementation, and milk consumption), while the dashed green line represents the diagonal reference line. All of models were evaluated using 200 bootstrap resampling iterations for internal validation. The optimism-corrected AUC values for the reduced model were 0.781 (95%CI=0.728~0.814) for overall prediction model 0.705 (95%CI=0.639~0.792) for Stratum 1 and 0.719 (95%CI=0.651~0.787) for Stratum 2. The diagonal reference line represents an AUC of 0.5 (no discrimination).

**Figure 2** ROC curves for the prediction model in Stratum 3. (A) presents the apparent ROC curves based on the development dataset, and (B) presents the bootstrap-corrected ROC curves derived from 200 resampling iterations. In both panels, the solid blue line represents the full model including all five perinatal/gestational factors (maternal education, intrauterine position, parity, vitamin AD supplementation, and milk consumption), while the dashed green line represents the diagonal reference line. The AUC values for the reduced model were 0.675 (95%CI=0.559~0.791) for the apparent performance and 0.660 (95%CI=0.518~0.801) after bootstrap correction. The diagonal green reference line represents an AUC of 0.5. The limited predictive performance in this stratum indicates that the perinatal/gestational factors have minimal discriminative value in Graf type IV hips, where disease severity appears to be the dominant determinant of treatment outcomes

### Table 5. Multivariable logistic regression analysis for the overall prediction model

| **Variable** | **β** | **SE** | **Wald χ²** | **p‑value** | **OR** | **95% CI** |
| --- | --- | --- | --- | --- | --- | --- |
| Graf classification  (Ref: Type IV) |  |  |  |  |  |  |
| Type D or III | 1.957 | 0.265 | 54.711 | < 0.001 | 7.081 | 4.215–11.894 |
| Type IIc | 2.286 | 0.279 | 67.250 | < 0.001 | 9.833 | 5.694–16.979 |
| Maternal education level (Ref: Middle school or below) |  |  |  |  |  |  |
| High school / Technical secondary school | 0.675 | 0.247 | 7.450 | 0.006 | 1.963 | 1.209–3.187 |
| Associate degree | 0.788 | 0.254 | 9.663 | 0.002 | 2.200 | 1.338–3.616 |
| Bachelor’s degree or above | 0.847 | 0.232 | 13.308 | < 0.001 | 2.332 | 1.480–3.677 |
| Intrauterine position  (Ref: Cephalic) |  |  |  |  |  |  |
| Non-cephalic | -1.033 | 0.229 | 20.359 | < 0.001 | 0.356 | 0.227–0.558 |
| Parity (ref: 1) |  |  |  |  |  |  |
| ＞1 | 0.895 | 0.234 | 14.648 | < 0.001 | 2.447 | 1.548–3.870 |
| Maternal vitamin AD supplementation during pregnancy (Ref: No) |  |  |  |  |  |  |
| Yes | 0.629 | 0.186 | 11.380 | < 0.001 | 1.875 | 1.301–2.702 |
| Maternal milk consumption during pregnancy  (Ref: None) |  |  |  |  |  |  |
| Occasional | 0.444 | 0.245 | 3.280 | 0.070 | 1.560 | 0.964–2.523 |
| Regular | 0.890 | 0.263 | 11.425 | < 0.001 | 2.435 | 1.453–4.079 |
| **Intercept** | −0.2771 | 0.553 | 24.072 | < 0.001 | — | — |

OR, odds ratio; CI, confidence interval; SE, standard error; ref, reference category. The outcome variable is Pavlik harness treatment success (success = 1, failure = 0). The model was internally validated using bootstrap resampling with 200 iterations; the optimism‑corrected C‑index was 0.788.

### Table 6. Multivariable logistic regression analysis for Stratum 1 (Graf type IIc)

| **Variable** | **β** | **SE** | **Wald χ²** | **p‑value** | **OR** | **95% CI** |
| --- | --- | --- | --- | --- | --- | --- |
| Maternal education level (Ref: Middle school or below) |  |  |  |  |  |  |
| High school / Technical secondary school | 0.632 | 0.430 | 2.163 | 0.141 | 1.882 | 1.209–3.187 |
| Associate degree | 0.498 | 0.414 | 1.447 | 0.229 | 1.645 | 0.731–3.699 |
| Bachelor’s degree or above | 0.972 | 0.411 | 5.067 | 0.018 | 2.644 | 1.182–5.911 |
| Intrauterine position  (Ref: Cephalic) |  |  |  |  |  |  |
| Non-cephalic | -0.781 | 0.445 | 3.085 | 0.079 | 0.458 | 0.192–1.095 |
| Parity (ref: 1) |  |  |  |  |  |  |
| ＞1 | 1.476 | 0.466 | 10.034 | 0.002 | 4.375 | 1.755–10.903 |
| Maternal vitamin AD supplementation during pregnancy (Ref: No) |  |  |  |  |  |  |
| Yes | 0.548 | 0.320 | 2.923 | 0.087 | 1.729 | 0.923–3.239 |
| Maternal milk consumption during pregnancy  (Ref: None) |  |  |  |  |  |  |
| Occasional | 0.209 | 0.411 | 0.259 | 0.611 | 1.232 | 0.551–2.757 |
| Regular | 0.634 | 0.451 | 1.976 | 0.160 | 1.885 | 0.779–4.563 |
| **Intercept** | -1.035 | 0.895 | 1.338 | 0.247 | — | — |

OR, odds ratio; CI, confidence interval; SE, standard error; ref, reference category. The outcome variable is Pavlik harness treatment success (success = 1, failure = 0). Stratum 1 includes patients with Graf type IIc. The model was internally validated using bootstrap resampling with 200 iterations; the optimism‑corrected C‑index was 0.707.

Table 7. Multivariable logistic regression analysis for Stratum 2

| **Variable** | **β** | **SE** | **Wald χ²** | **p‑value** | **OR** | **95% CI** |
| --- | --- | --- | --- | --- | --- | --- |
| Maternal education level (Ref: Middle school or below) |  |  |  |  |  |  |
| High school / Technical secondary school | 0.831 | 0.352 | 5.591 | 0.018 | 2.296 | 1.153–4.573 |
| Associate degree | 1.075 | 0.367 | 8.571 | 0.003 | 2.929 | 1.426–6.014 |
| Bachelor’s degree or above | 0.920 | 0.320 | 8.267 | 0.004 | 2.509 | 1.340–4.698 |
| Intrauterine position  (Ref: Cephalic) |  |  |  |  |  |  |
| Non-cephalic | -1.185 | 0.296 | 15.989 | ＜0.001 | 0.306 | 0.171–0.547 |
| Parity (ref: 1) |  |  |  |  |  |  |
| ＞1 | 0.878 | 0.335 | 6.864 | 0.009 | 2.407 | 1.248–4.643 |
| Maternal vitamin AD supplementation during pregnancy (Ref: No) |  |  |  |  |  |  |
| Yes | 0.712 | 0.267 | 7.128 | 0.008 | 2.038 | 1.208–3.436 |
| Maternal milk consumption during pregnancy  (Ref: None) |  |  |  |  |  |  |
| Occasional | 0.474 | 0.350 | 1.835 | 0.175 | 1.607 | 0.809–3.193 |
| Regular | 1.023 | 0.377 | 7.374 | 0.007 | 2.782 | 1.329–5.822 |
| **Intercept** | -0.828 | 0.709 | 1.331 | 0.249 | — | — |

OR, odds ratio; CI, confidence interval; SE, standard error; ref, reference category. The outcome variable is Pavlik harness treatment success (success = 1, failure = 0). Stratum 2 includes patients with Graf types D or III. The model was internally validated using bootstrap resampling with 200 iterations; the optimism‑corrected C‑index was 0.723.

**Figure 3** Calibration curve for the prediction model. (A) Overall prediction model, (B) presents Stratum 1 and (C) Stratum 2. Calibration of the prediction models was assessed using calibration plots comparing predicted versus observed probabilities of treatment success for the overall model and each stratified model (Stratum 1: Graf type IIc; Stratum 2: Graf types D/III). Bootstrap resampling with 200 iterations was performed to correct for overfitting optimism. The calibration slope (ideal value = 1) and calibration intercept (ideal value = 0) were calculated as quantitative measures of calibration. The Brier score was computed as a measure of overall prediction accuracy, with lower scores indicating better performance (a score of 0.25 represents a non‑informative prediction for binary outcomes). The x-axis represents the predicted probability of treatment success, and the y-axis represents the observed probability. The solid blue line represents the calibration curve derived from bootstrap resampling (200 iterations), and the dashed red line represents the ideal 45° reference line (perfect calibration). The shaded area represents the 95% confidence interval.

Table 8. Calibration metrics for the overall and stratified prediction models

| **Model** | **n** | **Calibration slope (95% CI)** | **Calibration intercept (95% CI)** | **Brier score** |
| --- | --- | --- | --- | --- |
| Overall | 792 | 0.952 (0.891–1.013) | −0.018 (−0.082 -0.046) | 0.182 |
| Stratum 1  (Graf IIc) | 306 | 0.941 (0.872–1.010) | −0.025 (−0.097 - 0.047) | 0.191 |
| Stratum 2  (Graf D/III) | 385 | 0.963 (0.905–1.021) | −0.011 (−0.079 - 0.057) | 0.175 |

**Figure 4** Decision curve analysis for the overall prediction model. Decision curve analysis (DCA) was performed to evaluate the clinical utility of the prediction models by quantifying the net benefit across a range of threshold probabilities for the overall model and each stratified model (Stratum 1: Graf type IIc; Stratum 2: Graf types D/III). The net benefit was calculated as: Net Benefit = (True Positives / n) − (False Positives / n) × (Threshold / (1 − Threshold)). The model‘s net benefit was compared with two reference strategies: (1) “Treat All” (intervening on all patients) and (2) “Treat None” (intervening on no patients). A model is considered clinically useful if it demonstrates a higher net benefit than both reference strategies across a clinically meaningful range of thresholds. The table presents the net benefit at selected threshold probabilities for the overall prediction model, compared with the “Treat All” strategy (intervening on all patients) and the “Treat None” strategy (intervening on no patients). Net benefit values are calculated as: Net Benefit = (True Positives / n) − (False Positives / n) × (Threshold / (1 − Threshold)). A positive net benefit at a given threshold indicates that the model provides clinical benefit over the “Treat None” strategy at that threshold. The model demonstrated a positive net benefit across threshold probabilities ranging from approximately 0 to 0.24, with the maximum net benefit observed at the lowest thresholds. Beyond a threshold of 0.24, the net benefit became negative, indicating limited clinical utility at higher decision thresholds.

Table 9. Net benefit at selected threshold probabilities for the overall and stratified prediction models

| **Threshold** | **Overall model** | **Stratum 1**  **(Graf IIc)** | **Stratum 2**  **(Graf D/III)** |
| --- | --- | --- | --- |
| 0.1 | 0.66 | 0.78 | 0.70 |
| 0.2 | 0.63 | 0.75 | 0.66 |
| 0.3 | 0.58 | 0.72 | 0.61 |
| 0.4 | 0.53 | 0.67 | 0.55 |
| 0.5 | 0.46 | 0.60 | 0.48 |
| 0.6 | 0.39 | 0.51 | 0.40 |
| 0.7 | 0.27 | 0.36 | 0.26 |
| 0.8 | 0.16 | 0.25 | 0.13 |

Table 10. Sensitivity analysis: Multivariable logistic regression model including age at treatment initiation as a covariate

| **Variable** | **OR** | **95% CI** | **P‑value** |
| --- | --- | --- | --- |
| Age at treatment initiation (per 7‑day increase) | 0.782 | 0.472–0.993 | < 0.001 |
| Graf D/III (vs.IV) | 4.403 | 2.04–7.883 | < 0.001 |
| Graf IIc (vs. IV) | 6.126 | 4.805–9.962 | < 0.001 |
| Maternal education:High school / Technical secondary school  (vs. Middle school or below) | 1.488 | 1.150–2.357 | 0.013 |
| Maternal education:Associate degree (vs. Middle school or below) | 2.017 | 1.541–2.635 | 0.007 |
| Maternal education:Bachelor’s degree or above (vs. Middle school or below) | 2.088 | 1.652–3.019 | < 0.001 |
| Intrauterine position: Non-Cephalic (vs. Cephalic) | 0.438 | 0.219–0.630 | < 0.001 |
| Parity: ＞1 (vs. 1) | 2.141 | 1.189–2.846 | < 0.001 |
| Maternal vitamin AD supplementation during pregnancy: Yes (vs. No) | 1.492 | 1.042–2.558 | 0.013 |
| Maternal milk consumption during pregnancy: Occasional (vs. None) | 1.266 | 0.956–1.784 | 0.091 |
| Maternal milk consumption during pregnancy: Regular (vs. None) | 2.040 | 1.232–3.215 | 0.005 |

Sensitivity analysis: When age at treatment initiation was added as a covariate to the multivariable model (Table S10), the associations between the perinatal/gestational factors and treatment outcomes remained largely unchanged, with overlapping 95% confidence intervals compared with the primary model (Table 2 in minuscript). Age at treatment initiation was itself significantly associated with treatment success (OR per 7-day increase: 0.782, 95% CI: 0.472–0.993, *P* < 0.001), confirming its established prognostic value. The ORs for Graf IIc and Graf D/III (vs. IV) were slightly attenuated after adjustment for age (from 6.126 to 4.403), suggesting that the effect of age is partially mediated through its association with disease severity. These findings confirm the robustness of our primary model and indicate that the effect of age at treatment initiation is largely mediated through Graf classification rather than acting as an independent confounder. OR, odds ratio; CI, confidence interval. The outcome variable is Pavlik harness treatment success (success = 1, failure = 0). Age at treatment initiation was entered as a continuous variable (per 7-day increase). The primary model (without age) is presented in Table 2. Age at treatment initiation was strongly correlated with Graf classification (Spearman’s r = −0.68; VIF > 5 when both variables were included in the same model). Therefore, age was not included in the primary model to avoid multicollinearity. This sensitivity analysis demonstrates that the associations between perinatal/gestational factors and treatment outcomes remained stable after adjustment for age, confirming the robustness of our primary findings. The slight attenuation of ORs for Graf IIc and D/III (vs. IV) after adjustment for age suggests that the effect of age on treatment success is partially mediated through its association with disease severity.

Table 11 TRIPOD Checklist: Prediction Model Development

| **Section/Topic** | **Item** | **Development or Validation?** | **Checklist Item** | **Reported on Page/Line** |
| --- | --- | --- | --- | --- |
| **Title and abstract** |  |  |  |  |
| Title | 1 | D; V | Identify the study as developing and/or validating a multivariable prediction model, the target population, and the outcome to be predicted. | Title  Line 1-3 |
| Abstract | 2 | D; V | Provide a summary of objectives, study design, setting, participants, sample size, predictors, outcome, statistical analysis, results, and conclusions. | Abstract  Line 20-54 |
| **Introduction** |  |  |  |  |
| Background and objectives | 3a | D; V | Explain the medical context (including whether diagnostic or prognostic) and rationale for developing or validating the multivariable prediction model, including references to existing models. | Introduction, Line 57-83 |
|  | 3b | D; V | Specify the objectives, including whether the study describes the development or validation of the model or both. | Introduction, Line 84-92 |
| **Methods** |  |  |  |  |
| Source of data | 4a | D; V | Describe the study design or source of data (e.g., randomized trial, cohort, or registry data), separately for the development and validation data sets, if applicable. | Methods  Line 139-147 |
|  | 4b | D; V | Specify the key study dates, including start of accrual; end of accrual; and, if applicable, end of follow-up. | Follow-up protocol  Line 114-129 |
| Participants | 5a | D; V | Specify key elements of the study setting (e.g., primary care, secondary care, general population) including number and location of centres. | Participants  Line 95-98 |
|  | 5b | D; V | Describe eligibility criteria for participants. | Participants  Line 98-104 |
|  | 5c | D; V | Give details of treatments received, if relevant. | Supplementary Materials,  Method S1 |
| Outcome | 6a | D; V | Clearly define the outcome that is predicted by the prediction model, including how and when assessed. | Statistical Analysis  Line 186-195 |
|  | 6b | D; V | Report any actions to blind assessment of the outcome to be predicted. | NA |
| Predictors | 7a | D; V | Clearly define all predictors used in developing or validating the multivariable prediction model, including how and when they were measured. | Methods,  Line 139-164 |
|  | 7b | D; V | Report any actions to blind assessment of predictors for the outcome and other predictors. | NA |
| Sample size | 8 | D; V | Explain how the study size was arrived at. | Supplementary Materials,  Method S2 |
| Missing data | 9 | D; V | Describe how missing data were handled (e.g., complete-case analysis, single imputation, multiple imputation) with details of any imputation method. | Participants  Line 98-104 |
| Statistical analysis methods | 10a | D | Describe how predictors were handled in the analyses. | Statistical Analysis  Line 166-195 |
|  | 10b | D | Specify type of model, all model-building procedures (including any predictor selection), and method for internal validation. | Statistical Analysis  Line 172-195 |
|  | 10c | V | For validation, describe how the predictions were calculated. | Statistical Analysis  Line 190-193 |
|  | 10d | D; V | Specify all measures used to assess model performance and, if relevant, to compare multiple models. | Statistical Analysis  Line 172-195 |
|  | 10e | V | Describe any model updating (e.g., recalibration) arising from the validation, if done. | NA |
| Risk groups | 11 | D; V | Provide details on how risk groups were created, if done. | NA |
| Development vs. validation | 12 | V | For validation, identify any differences from the development data in setting, eligibility criteria, outcome, and predictors. | NA |
| **Results** |  |  |  |  |
| Participants | 13a | D; V | Describe the flow of participants through the study, including the number of participants with and without the outcome and, if applicable, a summary of the follow-up time. A diagram may be helpful. | Figure 1 |
|  | 13b | D; V | Describe the characteristics of the participants (basic demographics, clinical features, available predictors), including the number of participants with missing data for predictors and outcome. | Results  Line 198-205 |
|  | 13c | V | For validation, show a comparison with the development data of the distribution of important variables (demographics, predictors and outcome). | NA |
| Model development | 14a | D | Specify the number of participants and outcome events in each analysis. | Results  Line 206-214  Table2 |
|  | 14b | D | If done, report the unadjusted association between each candidate predictor and outcome. | NA |
| Model specification | 15a | D | Present the full prediction model to allow predictions for individuals (i.e., all regression coefficients, and model intercept or baseline survival at a given time point). | Supplementary Materials,  Table S5-S7 |
|  | 15b | D | Explain how to use the prediction model. | Results  Line 281-291 |
| Model performance | 16 | D; V | Report performance measures (with CIs) for the prediction model. | Table 1-4,  Table S5-S10 |
| Model-updating | 17 | V | If done, report the results from any model updating (i.e., model specification, model performance). | NA |
| **Discussion** |  |  |  |  |
| Limitations | 18 | D; V | Discuss any limitations of the study (such as nonrepresentative sample, few events per predictor, missing data). | Discussion  Line 475-495 |
| Interpretation | 19a | V | For validation, discuss the results with reference to performance in the development data, and any other validation data. | NA |
|  | 19b | D; V | Give an overall interpretation of the results, considering objectives, limitations, results from similar studies, and other relevant evidence. | Conclusion  Line 496-505 |
| Implications | 20 | D; V | Discuss the potential clinical use of the model and implications for future research. | Discussion  Line 453-474 |
| **Other information** |  |  |  |  |
| Supplementary information | 21 | D; V | Provide information about the availability of supplementary resources, such as study protocol, Web calculator, and data sets. | Supplementary Materials |
| Funding | 22 | D; V | Give the source of funding and the role of the funders for the present study. | Funding  Line 513-514 |

Table 12 Comparison of key baseline characteristics between included and excluded patients

| Factors | Included（n=792） | Excluded（n=178） | t / χ^2^ | p value |
| --- | --- | --- | --- | --- |
| Gender | 1 |  | 1.891 | 0.169 |
| Female | 716（90.4） | 153（87.1） |  |  |
| Male | 76（9.6） | 23（12.9） |  |  |
| Graf classification |  |  | 0.038^a^ | 0.981 |
| IIc | 306（38.6） | 70（39.3） |  |  |
| D or III | 385（48.6） | 86（48.3） |  |  |
| IV | 101（12.8） | 22（12.4） |  |  |
| Age at treatment initiation | 70.5 ± 35.0 days | 68.9 ± 27.6 days | 0.663 | 0.508 |

*^a^* Calculated using the Kruskal-Wallis H test.
